# Evaluation of blood reservation and use for caesarean sections in a tertiary maternity unit in south western Nigeria

**DOI:** 10.1186/1471-2393-10-57

**Published:** 2010-09-23

**Authors:** Oluwarotimi I Akinola, Adetokunbo O Fabamwo, Adetokunbo O Tayo, Kabiru A Rabiu, Yussuf A Oshodi, Chioma A Onyekwere

**Affiliations:** 1 Department of Obstetrics and Gynaecology, Lagos State University Teaching Hospital, Ikeja, Lagos State, Nigeria

## Abstract

**Background:**

Haemorrhage from obstetric causes is the most common cause of maternal mortality in the developing world. Prevention of mortality from haemorrhage will necessarily involve prompt blood transfusions among other life saving measures. There are however limited stocks of fresh or stored blood in many health care facilities in Sub Saharan Africa. Caesarean section has been identified as a common indication for blood transfusion in obstetrics practice and its performance is often delayed by non availability of blood in our centre. An evaluation of blood reservation and use at caesarean sections in a tertiary maternity unit in Lagos, south western Nigeria should therefore assist in formulating the most rational blood transfusion policies.

**Methods:**

Case records of 327 patients who had elective and emergency caesarian sections at the Lagos State University Teaching Hospital between 1^st ^October and 31^st ^December 2007 were reviewed. Data pertaining to age, parity, booking status, type and indication for Caesarean section, pre- and post-operative packed cell volume, blood loss at surgery, units of blood reserved in the blood bank, unit(s) of blood transfused and duration of hospital stay was extracted and the data analysed.

**Results:**

There were 1056 deliveries out of which 327 (31%) were by Caesarean section. During the study period, a total of 654 units of blood were reserved in the blood bank and subsequently made available in theatre. Out of this number, only 89 (13.6%) were transfused to 41 patients. Amongst those transfused, twenty-six (54%) were booked and 31 (75.6%) had primary caesarian section. About 81% of those transfused had emergency caesarean section. The most common indication for surgery among those transfused were placenta praevia (9 patients with 21 units of blood) and cephalo-pelvic disproportion (8 patients with 13 units).

**Conclusion:**

Even though a large number of units of blood was reserved and made available in the theatre at the time of operation, majority of the patients operated did not need blood transfusion. Provision of a mini- blood bank within the obstetric unit and careful patient categorization will ensure timely availability of blood for surgery without necessarily tying down stock in the central blood bank.

## Background

Peripartal haemorrhage is still the leading cause of maternal and fetal morbidity and mortality in developing countries [1]. Despite advances in the prevention, diagnosis and treatment, massive blood loss during pregnancy and delivery remains a threat and therefore, prevention of maternal mortality involves prompt blood transfusions among other life saving measures to attain the fifth millennium development goal [2].

Caesarian delivery is often performed in young patients who are free of serious cardiovascular and pulmonary diseases. The procedure has been identified as a common indication for blood transfusion in obstetric practice because it involves risk of major intra-operative blood loss [3]. Its performance is often delayed by non-availability of blood [4,5]. The increased blood volume associated with normal pregnancy typically accommodates the obligatory blood loss that occurs during vaginal or caesarian delivery [6]. However, in some patients, blood loss may overwhelm compensatory mechanisms and result in hypovolaemia and shock with a significant threat to both the mother and fetus [7]. Transfusion practices have changed during the last two decades because of acute awareness of the increased risks associated with blood transfusion as well as an improved knowledge of the patho-physiologic mechanism of oxygen transport and tissue oxygenation [3].

In our centre, anaesthetists usually request for a minimum of two units of cross-matched blood for cases of caesarean section irrespective of the pre-operative haematocrit. Most of these cross-matched pints of blood are seldom used. The blood gets tied down and is unavailable for other users. Unnecessary blood reservation, apart from the consideration of cost, may result in apparent blood scarcity especially in facilities where blood is in short supply. This then denies those patients who really need blood for life saving interventions [8].

There is evidence to suggest that the attitude to blood preservation and use is fairly similar in this subregion [8]. The need to focus on preserving the blood supply, increasing availability, ensuring rational reservation and use, enhancing its safety and decreasing cost without compromising the quality of care informs the necessity for this study. We therefore undertook an audit of blood reservation and transfusion practices for caesarean section at this centre with a view to recommending modifications wherever it is found to be suboptimal.

## Methods

This was a retrospective descriptive study conducted between 1^st ^October and 31^st ^December 2007 in the Obstetrics and Gynaecology Department of the Lagos State University Teaching Hospital (LASUTH), Ikeja. The study protocol was approved by the Research and Ethics Committee of the institution.

Case records of patients who had caesarean delivery during the period were reviewed. Data were obtained on age, parity, booking status, type of and indication for caesarian section, blood loss at surgery and duration of hospital stay. Other information extracted include pre and post operative haematocrit, units of blood reserved at the blood bank and units of blood actually transfused. Booked cases were those that registered and were receiving antenatal care in the department of Obstetrics and Gynaecology, LASUTH, while unbooked cases were those that were brought in as emergency outside LASUTH even though they might have received antenatal care elsewhere.

Surgery was performed by experienced residents and consultants according to standardized protocol [9] and placenta were delivered by controlled cord traction except where this was difficult and manual removal performed. Blood loss was estimated by counting the number of soaked abdominal packs, gauzes, measurement of blood volume in the vagina after caesarean section and visual estimation of blood staining of the theatre bedspread.

Data obtained were analyzed with SPSS version XIV (Chicago Illinois). Variables were summarized using frequency, mean and standard deviation. The Chi-square test and Student's t-test were used to test for associations between variables as appropriate. *P *value of less than 0.05 was considered statistically significant, confidence level was set at 95%.

## Results

Three hundred and twenty seven patients had caesarean delivery amongst 1056 parturients during the study period giving a caesarian section rate of 40%. Six hundred and fifty four units of blood were cross-matched for the procedure but only 89 units (13.6%) were transfused giving cross-matched: transfusion ratio of 7.4:1.

Forty-one (12.5%) of those that had Caesarean section were transfused. Table 1 shows the characteristics of the patients. Two-hundred and twenty-one (67.5%) patients were booked, out of whom 26 (11.7%) were transfused whereas 15 (14.2%) among the 106 unbooked patients were transfused.

**Table 1:** Characteristics of patients that had caesarian section and blood use

Parameters	Total C/S	No. transfused	Units	
**Booking status**				
Booked	221 (67.5%)	26 (11.7%)	54 (61%)	P = 0.09
Unbooked	106 (32.5%)	15 (14.2%)	35 (39%)	
				
**Type of C/S**				
Elective	82 (25%)	8 (9.8%)	19 (21%)	P < 0.05
Emergency	245 (75%)	33 (13.5%)	70 (79%)	
				
Primary C/S	232 (71%)	33 (14.2%)	72 (81%)	P < 0.05
Repeat C/S	95 (29%)	8 (8.4%)	17 (19%)	
				
**Total**	327	41	89	

Elective caesarian section was performed on 82 patients out of whom 8 (9.8%) were transfused compared to 33 (13.5%) that were transfused among those that had emergency caesarian section (P < 0.05). Two hundred and thirty-two patients had primary caesarean section out of whom 33 were transfused (14.2%) compared to only 8 (8.4%) that were transfused among the 95 patients who had repeat caesarian section.

Table 2 depicts indications for caesarean section with reference to the frequency and blood transfusion practice. Indication for surgery among those transfused were placenta praevia (9 patients) who had 21 units of blood, Cephalo-pelvic disproportion (8 patients) with 13 units, previous caesarian section (4 patients) with 10 units, Sickle cell disease Hb SS (3 patients) with 7 units while eclampsia and fetal distress accounted for 2 patients who had transfusion with 4 and 3 units respectively.

**Table 2:** Indications for caesarean section and number transfused

Indication	Frequency	No. transfused	Units received
CPD*	61	6	9 (10.1%)
Previous Scar	52	7	16 (18%)
Fetal distress	34	3	5 (5.6%)
Placenta praevia	22	9	21(23.6%)
Breech	20	1	1 (1.1%)
Pre-eclampsia/eclampsia	19	2	4 (4.5%)
PL^#^/Obst^##^. Labour	17	3	7 (7.9%)
Failed Induction	12	1	1 (1.1%)
BOH**	9	-	-
Abnormal CTG	9	-	-
Malpositioning	8	1	1 (1.1%)
Multiple pregnancy	8	2	5 (5.6%)
Cervical dystocia	8	-	-
PMTCT^+^	8	1	4 (4.5%)
Malpresentation	5	1	1 (1.1%)
HbSS***	3	2	7 (7.9%)
Abruptio placenta	3	2	7 (7.9%)
Others	29	-	-

			89 (100%)

* Cehalopelvic disproportion ^# ^Prolonged labour

** Bad obstetrics history ^## ^Obstructed labour

^+ ^Prevention of mother to child transmission *** Sickle cell anaemia

Table 3 compares some parameters between transfused and non-transfused patients. There was no statistically significant difference between their mean age (30.2 ± 5.18 years) and (30.4 ± 4.93). Similarly, there was no significant difference in the parity of those transfused (range 0-4, median 1) and non-transfused (range 0-4, median 1). However, estimated blood loss (EBL) at surgery, pre-operative and post- operative haematocrit demonstrated statistically significant differences between the two groups. The mean EBL for the transfused subjects was 848.3 ± 736.2 ml compared to 537.5 ± 294.2 ml in the non-transfused subjects (P < 001). The mean pre-operative haematocrit in those transfused was 27.2 ± 6.1 percent compared to 33.6 ± 5.3 percent obtained in the non-transfused subjects (P < 001). Similarly, the mean post-operative haematocrit in those transfused was 26.6 ± 5.7 percent compared to 31.1 ± 5.1 percent obtained in the non-transfused subjects (P < 001).

**Table 3:** Comparison between those transfused and non-transfused C/S

Parameter	Non-transfused		Transfused		P value
	Mean	(S/D)	Mean	(S/D)	
Age	30.4	(4.9)	30.2	(5.2)	P = 0.17
Parity	1.02	(1.1)	1.07	(0.9)	P = 0.13
Blood loss	537.5	(294.2)	848.3	(736.2)	P < 0.01
Pre-op PCV	33.6	(5.3)	27.2	(6.1)	P < 0.01
Post-op PCV	31.1	(5.1)	26.6	(5.7)	P < 0.001
Hospital stay	8.3	(3.3)	10	(4.0)	P = 0.06
Units transfused	---		2.2	(1)	

Table 4 depicts the unit(s) of blood transfused and the frequency. It was observed that seven patients had one unit transfusion (17%), 25 patients were transfused with two units of blood (61%) and 5 had three units of blood transfusion (12.2%). Others include 3 patients that had four units of blood (7.3%) and one who had five units of blood transfused (2.5%).

**Table 4:** Pattern of transfusion and frequency of usage

Unit of blood	No. of patients (%)	Units transfused
1	7 (17.0%)	7
2	25(60.9%)	50
3	5(12.2%)	15
4	3(7.3%)	12
5	1(2.5)	5

Total	41(100%)	89

## Discussion

The increasing use of surgery for childbirth and subsequent need for blood transfusion together with patient's reluctance to receive homologous transfusion poses a challenge to the obstetricians and anaesthetists [10]. Although, improvement in obstetrics surgical techniques and practice may have decreased the use of homologous blood transfusion at the time of caesarean section, the risk of requiring blood transfusion is still significant especially in high risk cases [11]. Certain trends or consideration have contributed to decreasing transfusion rate. These include physician's acceptance of lower peri-operative haemoglobin concentration or haematocrit levels, reduced patient's willingness to accept the risk of transmission of blood borne infectious agents, more restrictive indications for blood transfusion and the fact that the obstetrics population is largely young and healthy [12].

The caesarean section rate in this study was 40% which is very high compared to 10-15% in the United States[13] and 5-21.8% reported in Sub-saharan Africa [14]. However, the World Health Organization suggested a caesarian section rate of 5-15% in any facility [15]. This high incidence has been an issue of international health concern although most cases in this study were emergencies with genuine indications. Our facility also serves as one of 3 tertiary referral centres for Lagos metropolis with a population of about 15 million inhabitants.

The transfusion rate among the patients that had caesarean delivery was 12.5%. This is consistent with transfusion rate of 1-14% as suggested by review of literature for blood transfusion following caesarean section [11]. The blood transfusion rate in this study is higher than 4.9% and 5.4% reported by Duthie et al [16] and Rouse et al [17] but significantly lower than 23.5% and 25.2% reported by Rainaldi et al [10] and Ozumba et al [18].

Considering the demographic characteristic of patients who had blood transfusion and those who did not, the age, parity and booking status were not significantly associated with increased risk of blood transfusion. This is contrary to the findings of Imarengiaye et al [19] who reported a six fold risk of blood transfusion in unbooked cases and might be a reflection of some degree of antenatal care even in the 'unbooked' patients in a cosmopolitan setting as ours. However, emergency caesarean section was found to increase the risk of transfusion as 13.5% of patients in this category were transfused compared to 9.8% of those that had elective surgery. This finding is consistent with the report by Tolby and Scott [20] who found a statistically significant risk of transfusion in their subjects undergoing emergency caesarean section.

Of the 232 subjects that had primary caesarean section, 14.2% of them were transfused compared to 8.4% of the 95 subjects that had repeat surgery. This was found to be statistically significant (P < 0.05). Our finding is in contrast with that of Imarengiaye [19] who found significant risk of transfusion with repeat caesarean section. However, this increased rate of transfusion with primary surgery in our study may be because of a preponderance of cases related to prolonged labour secondary to cephalo-pelvic disproportion. In these cases, there is usually post-operative uterine atony due to muscle fatigue in addition to low pre-operative haematocrit among the unbooked emergencies. As noted in this study, cephalo-pelvic disproportion was found to be the most common indication for emergency caesarian section in most series [18,21]

The highest transfusion rate was seen in cases of placenta praevia with 9 patients receiving a total of 21 units of blood transfusion. This accounts for 41% of total patients with placenta praevia as the indication for surgery compared to 59.1% similar subjects transfused in the Ozumba study [18]. Pregnancies complicated by placenta praevia are noted for increased blood loss and transfusion at surgery. Factors responsible include repeated ante-partum haemorrhage which may lower the haematocrit, thus putting the patient at a point close to transfusion trigger. Similarly, the low-lying placenta may provoke increased and uncontrollable intra-operative haemorrhage necessitating blood transfusion [19]. Other indications for caesarian section with associated risk of transfusion in this study include previous uterine scar, prevention of mother to child transmission of human immune deficiency virus, obstructed labour, multiple pregnancy, abruptio placenta, haemoglobinopathy and pre-eclampsia/eclampsia. Most of these factors have been corroborated by other authors [17-19,22,23].

The estimated blood loss at surgery in this study was significantly associated with increased risk of blood transfusion. The mean EBL among patients transfused was 848.3 ± 736.2 mls compared to 537.5 ± 294.2 mls in the non-transfused patients (P < 0.01). The corresponding EBL values reported by Imarengiaye et al [19] was 1310.8 ±991.8 mls and 592.5 ± 181.7 (P = 0.001) for transfused and non-transfused subjects respectively. Others authors also found strong association between intra-operative blood loss and risk of blood transfusion at caesarian section [3,11,17-19]. The role of anaemia as represented by pre- and post-operative haematocrit level was also found to be significantly associated with increased transfusion risk in this study (P < 0.01). This association was reported in other works [17-19]. Though a transfusion haematocrit threshold of 30% or less has been suggested as appropriate [24], the mean pre-transfusion haematocrit in this study was 27.2%. While women who underwent caesarian section may tolerate post-operative haematocrit of 20% without significant complications, transfusion with red blood cells may be appropriate when the haematocrit is 21-30% if there is active bleeding or cardio-pulmonary disturbance [25].

The breakdown of units of blood transfused revealed that 7 (17%) of the subjects received one unit of blood. This is slightly higher than 13.3% reported by Imarengiaye et al [19] but significantly lower than 43.1% and 68.2% of one unit transfusion reported by Ozumba et al [18] and Khan et al [23]. The latter authors opined that those transfusions were unnecessary and the patients could have survived with plasma expanders instead of one unit of blood transfusion with its attendant risks and complications.

Moreover, several professional societies suggested that the decision to transfuse a specific patient should take into consideration many factors other than specific haemoglobin concentration [26-29]. While the American College of Physicians suggested that even in a symptomatic patient, one unit transfusion may be sufficient, recent guidelines by the British Committee for standard in haematology recommended that if otherwise stable, two units of red cells should be transfused [27-29]. However, the goal of transfusion can be achieved in certain instances with a single unit of blood transfusion with an interim assessment to determine the need for second unit. Therefore, the need for the well established practice of two unit transfusion regardless of the level of anaemia is questionable.

The short duration of this study and its cross sectional nature are significant limitations as it might not reflect long term practice and trends and certainly could not assess the long term effect of blood transfusion policy on subsequent health of the subjects [30]

Since only 89 of all 654 units cross-matched blood was used, this gave a cross match transfusion ratio of 7.4:1. It also indicated that only 12.5% of cross-matched blood was used. The cross-match transfusion ratio is a method of evaluating the efficiency of blood bank ordering practice [17]. The cost of cross-matching a unit of blood in our facility is three thousand naira (#3000:00 = 20 USD) and the cost of cross-matching for the unused 565 units of blood is #1,695,000 (11,300 USD). This amount represents a large wasteful financial expenditure and possibly waste products if the blood was not returned on time before denaturation sets in. Current literature advocates type and screen as a safe alternative to pre-operative type and cross-match for procedures requiring less than one unit per case [31]. Advocates of pre-operative type and screen policy for obstetric procedures claimed substantial savings as a result, suggesting that this policy could substantially reduce cost without harm to the patient. This policy change includes defining criteria for cross-matching patients with high risk for bleeding as listed earlier from identified risk factors for transfusion.

However, if the clinical circumstances of a low risk patient who initially had a hold clot order changes putting the patient at high risk, the blood bank is notified immediately. With this approach, delay in rendering care regarding transfusion need not arise as blood group O rhesus negative cross-matched blood could be given in the interim pending the availability of fully cross-matched blood.

It is desirable to revisit the blood banking order with respect to rational blood use and reservation for caesarian section. In the absence of significant risk factors for haemorrhage, routine pre-operative type and cross-match does not enhance patient's care, substantially increase cost and should be eliminated [32]. However, hold clot order for low risk patients, availability of blood group O rhesus negative cross-matched blood in a mini-blood bank attached to the obstetrics unit and careful categorization with cross-matched blood for high risks are viable alternatives that reduce cost and make utilization of scarce resources efficient.

## Conclusion

This study has shown that even though a large number of units of blood was reserved and made available in the theatre at the time of caesarean section, majority of the patients operated did not need blood transfusion, resulting in a large cross-match transfusion ratio and consequently a large waste of financial, laboratory and blood bank resources. In developing countries with limited availability of blood and blood products, provision of a mini-blood bank within the obstetric unit and careful patient categorization may probably ensure timely availability of blood for surgery without necessarily tying down stock in the central blood bank.

## Competing interests

The authors declare that they have no competing interests.

## Authors' contributions

OIA conceived the study and participated in its design and co-ordinated the data collection, analysis and wrote the first draft of the paper. AOF, AOT, KAR, YAO and CAO participated in the study design, data collection, analysis and helped to draft the manuscript. All authors read and approved the final manuscript.

## Pre-publication history

The pre-publication history for this paper can be accessed here:

http://www.biomedcentral.com/1471-2393/10/57/prepub
